# Reply to Comment on Roelofs, K.A.; et al. “Detecting Progression of Melanocytic Choroidal Tumours by Sequential Imaging: Is Ultrasonography Necessary?” *Cancers* 2020, *12*, 1856

**DOI:** 10.3390/cancers13061320

**Published:** 2021-03-16

**Authors:** Kelsey A. Roelofs, Roderick O’Day, Lamis Al Harby, Gordon Hay, Amit K. Arora, Mandeep S. Sagoo, Bertil Damato

**Affiliations:** 1 Ocular Oncology Service, Moorfields Eye Hospital, London EC1V 2PD, UK; roelofs@ualberta.ca (K.A.R.); roderick.oday@gmail.com (R.O.); lamis.alharby1@nhs.net (L.A.H.); gordon.hay3@nhs.net (G.H.); amit.arora@nhs.net (A.K.A.); mandeep.sagoo1@nhs.net (M.S.S.); 2 Department of Ophthalmology, Ocular Oncology Clinic, Royal Victorian Eye and Ear Hospital, Melbourne 3002, Australia; 3 NIHR Biomedical Research Centre for Ophthalmology at Moorfields Eye Hospital and University College London Institute of Ophthalmology, London EC1V 9EL, UK; 4 Nuffield Laboratory of Ophthalmology, University of Oxford, London OX3 9DU, UK

We thank Capasso et al. for their comment on our recent publication [1,2]. They have highlighted a very interesting topic, that being the role of internal reflectivity and internal vascularity in the monitoring of choroidal melanocytic tumors.

As Aristotle once noted, ‘the whole is greater than the sum of its parts,’ and particularly in ocular oncology where histopathological diagnosis is often impractical, careful consideration of the whole picture is important. To this end, we would like to firstly emphasize the importance of multimodal imaging, particularly in cases where ultrasonography is not performed. The purpose of our study was not to determine the usefulness of ultrasonography, but rather to determine if the sum of findings on multimodal imaging could compensate adequately in order to detect progression of choroidal melanocytic lesions in cases where echography is not performed. In our series, 100% of lesions categorized as MOLES 1 or 2 would have had progression detected without the need for ultrasonography. Therefore, our data show that in these lesions, the sensitivity for detecting progression is not affected by eliminating echography, regardless of what additional information it may or may not contribute.

To address a few of the specific concerns raised, Capasso et al. refer to case 81, whose tumor had a MOLES score of 4 and whose only sign of tumor progression was an increase in thickness. Based on the MOLES recommendations, a score of 4 is indicative of ‘probable melanoma’ and should trigger referral to an ocular oncologist in the first instance. Neither the data presented in our paper, nor the conclusions we have drawn from them, suggest that lesions with a MOLES score of 4 should be followed without ultrasonography.

While we agree with Capasso that in certain circumstances, echographic findings including intra-tumoral vascularity and internal reflectivity, can be helpful, the referenced paper by Ossoinig [3], published in 1979, is a review article on standardized echography and provides no data to support these conclusions. We have thoroughly reviewed the literature and were unable to find any scientific evidence to support that changes in internal reflectivity and/or intra-tumoral vascularity reliably identify progression of choroidal nevi to melanoma.

In our paper, we have advocated that MOLES 1 and 2 can be safely monitored without the need for ultrasonography. This conclusion is supported by the objective, scientific evidence presented in our paper. In addition, there are several reasons why this conclusion is a reasonable one. In the majority of cases, lesions that are classified as MOLES 1 and 2 have a thickness less than 2 mm, and therefore, many are too thin to accurately and reliably assess internal reflectivity and intrinsic vascularity on ultrasonography. Moreover, the thickness of these relatively small lesions is perhaps more accurately measured with OCT rather than ultrasonography [4].

Capasso et al. have also raised several concerns about the MOLES scoring system itself, which was designed to help non-experts estimate the likelihood of malignancy in melanocytic choroidal tumours according to mushroom shape, orange pigment, large size, enlargement and subretinal fluid, without the need for ultrasonography; however, their points are based on personal experience rather than objective scientific evidence. In contrast, a study of 222 patients at the Naevus Clinic of Moorfields Eye Hospital found that all 81 tumours diagnosed by the oncologist as melanoma had a MOLES score >2 (100% sensitivity) whereas 135/141 (96%) of tumours diagnosed as naevus had a MOLES score <3 [5]. Another study, also at Moorfields Eye Hospital, showed that all but one of 451 tumours treated for choroidal melanoma had a MOLES score >2 with none having a score <2 [1,2]. We acknowledge that further studies are needed to evaluate the use of MOLES in a variety of situations, because its success depends on the ability of clinicians to accurately identify various features, including orange pigment.

In summary, the results of our study contradict the opinion of Capasso et al. that safe monitoring of melanocytic choroidal tumours invariably requires standardized echography. We acknowledge the value of ultrasonography in the sub-specialty practice of ocular oncology; however, the purpose of this report was to identify lesions that can safely be followed with widely available multi-modal imaging. Finally, the significant benefit to patients in omitting echography in appropriate cases should not be overlooked or minimized. Reliance on more widely available imaging makes tele-consultations possible, thereby minimizing patient travel to sub-specialty centres where equipment and expertise for ultrasonography are available.

We respect the personal experience and opinion of Capasso et al. and appreciate the opportunity to further clarify the purpose and findings of our study. Although concluding that ultrasonography does not appear to be a universally required component of choroidal nevus monitoring perhaps breaks with tradition, it is our hope that the sum of our collective scientific inquiries will allow us to provide increasingly evidence-based care to our future patients.
